# Robust Enhancement of Lentivirus Production by Promoter Activation

**DOI:** 10.1038/s41598-018-33042-5

**Published:** 2018-10-11

**Authors:** Naoto Suzuki, Takeshi Yoshida, Hiroaki Takeuchi, Ryuta Sakuma, Sayaka Sukegawa, Shoji Yamaoka

**Affiliations:** 1 Department of Molecular Virology, Tokyo Medical and Dental University (TMDU), Tokyo, Japan; 2 Present Address: Medical Affairs Unit, ViiV Healthcare K.K., 1-8-1 Akasaka, Minato-ku, Tokyo, 107-0052 Japan; 3 Present Address: Department of Infectious Diseases and Immunology, Clinical Research Center, National Hospital Organization Nagoya Medical Center, Nagoya, Japan

## Abstract

Lentiviral vectors are a valuable tool to deliver exogenous genes for stable expression in cells. While much progress has been made in processing lentiviral vector-containing culture medium, it remains to be explored how the production of lentiviral vector from producer cells can be increased. We initially found that co-expression of the SPRY domain-containing SOCS box protein 1 (SPSB1) promotes the production of human immunodeficiency virus type 1 (HIV-1) and lentiviral vector with increased expression of the Gag and envelope proteins and activation of the HIV-1 LTR and CMV promoter. The presence of AP-1, NF-κB and CREB/ATF recognition sites in these promoters prompted us to utilize human T-lymphotropic virus type 1 (HTLV-1) Tax for lentiviral vector production because Tax activates all these transcription factors. Co-expression of a small amount of Tax markedly increased both the expression of viral structural proteins in producer cells and release of lentiviral vector particles, resulting in a more than 10-fold enhancement of transduction efficiency. Of note, the Tax protein was not detected in the lentiviral vector particles concentrated by ultracentrifugation, supporting the safety of this preparation. Collectively, these results indicate that promoter activation in producer cells represents a promising approach to preparing high-titer lentiviral vectors.

## Introduction

Lentiviral vectors derived from human immunodeficiency virus type 1 (HIV-1) are a valuable tool to transduce dividing and non-dividing cells with exogenous genes both *in vitro* and *in vivo*. The safety and convenience of lentiviral vectors have been improved over time by various different procedures^1^. Vesicular stomatitis virus glycoprotein (VSV-G)-pseudotyped lentiviral vectors are commonly produced in HEK293T cells under the control of the human cytomegalovirus (CMV) immediate early promoter and can mediate very efficient transduction into a wide range of cells^2^. Recent clinical applications of lentiviral vectors targeting hematopoietic stem cells and T cells have proven quite successful^3^. *In vivo* experiments and transduction of non-dividing cells often require a high-titer of the lentiviral vector, which has to be manufactured in large-scale cultures, concentrated by ultracentrifugation, and then purified by chromatography and ultrafiltration^4^. This procedure currently represents a major obstacle because of the high cost for vector preparation using large quantities of plasmids and cell culture materials as well as equipment for concentration and purification. While much effort has been expended to improve the procedures for concentration and purification of lentiviral vectors from culture supernatants, relatively few reports have described simple methods for enhancing the actual production of the vector. One showed that blocking innate antiviral responses increased viral vector production^5^ and another showed that stable expression of an immune response protein, Siglec-9, in producer cells enhanced lentiviral vector production by raising the transfection efficiency^6^. Other investigators have explored the addition of caffeine^7^ or sodium butyrate^8^ to the culture medium to increase the titer of lentiviral vector. However, further improving the production of lentiviral vectors remains a pressing issue for high-titer lentiviral vector preparation.

HIV-1 requires many host factors for its production^9^. Thus, ABCE1 promotes Gag multimerization and assembly^10^; ESCRT proteins are required for viral budding^11^; suppressor of cytokine signaling 1 (SOCS1) binds to the HIV-1 Pr55 Gag polyprotein and enhances its stability and trafficking, resulting in the efficient production of HIV-1 particles^12^; the microtubule network plays an important role in SOCS1-mediated HIV-1 Gag transport and virus particle formation^13^; and SOCS1 physically interacts with rhesus macaque TRIM5α (RhTRIM5α), counteracting its restriction at the late phase of HIV-1 replication^14^. RhTRIM5α is a well-known restriction factor for HIV-1 that potently blocks HIV-1 infection^15^. RhTRIM5α is composed of Really Interesting New Gene (RING), B-box 2, coiled-coil and SPRY domains, of which the latter is responsible for its recognition of the HIV core and is essential for the restriction^16^.

In our studies on host factors regulating HIV-1 replication, we became interested in the SPRY domain-containing SOCS box protein (SPSB) family proteins because they possess both SOCS Box and SPRY domains in a single molecule. SPSB family proteins recruit E3 ubiquitin ligase complexes and are thought to act as substrate recognition modules to mediate polyubiquitination. SPSB1, 2 and 4 were reported to mediate the binding between E3 ubiquitin ligase complexes and inducible nitric-oxide synthase (iNOS) to regulate iNOS degradation by the proteasome^17^. SPSB3 was reported to form E3 ubiquitin ligase complexes and mediate the polyubiquitination of a zinc finger transcription factor, SNAIL^18^. Tax protein of human T-lymphotropic virus type 1 (HTLV-1) is a *trans*-acting protein that promotes HTLV-1 LTR-driven transcription and activates cellular transcription factors including cAMP response element-binding protein (CREB)/activating transcription factor (ATF), nuclear factor-κB (NF-κB) and activator protein-1 (AP-1)^19^. In the present study, we demonstrate that both SPSB1 and HTLV-1 Tax enhance CMV promoter-driven gene expression in HEK293T cells and have exploited this finding for lentiviral vector production.

## Results

### SPSB1 increases production of HIV-1 and lentiviral vectors

**Figure 1:**
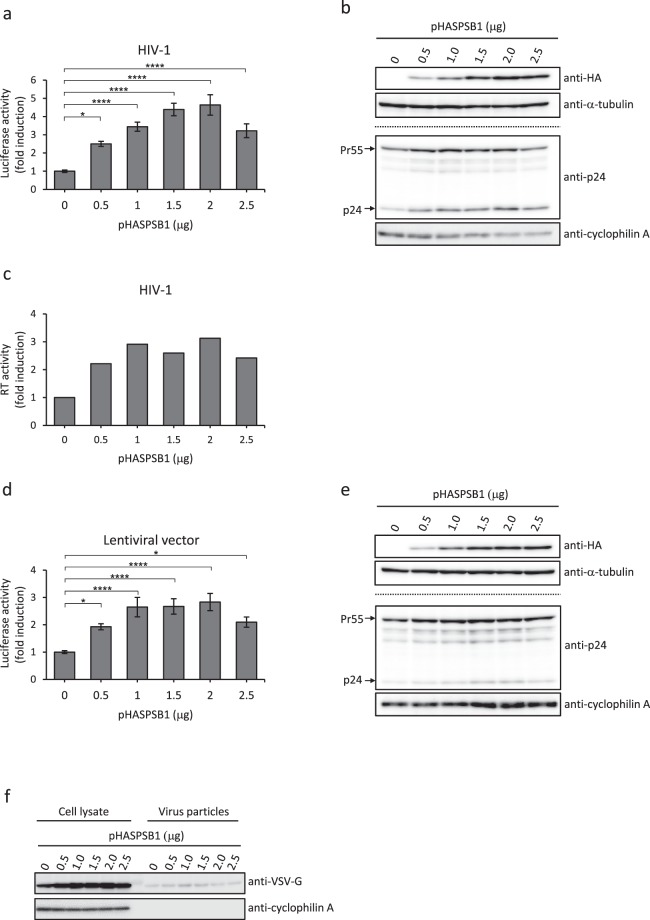
SPSB1 increases production of HIV-1 and lentiviral vector. (**a**) Approximately 1.5 × 10^6^ HEK293T cells were co-transfected with 1 μg of pNL4-3 and the indicated amounts of pHASPSB1 and/or pIRES-PURO empty vector (EV) so that the effector plasmids totaled 2.5 μg. HIV-1-containing culture supernatants of transfected cells were harvested and used to infect TZM-bl cells. The luciferase activity was normalized against the protein concentration and the results are shown as fold-increase compared to the control (0 μg of pHASPSB1). (**b**) Transfected HEK293T cells shown in Fig. 1a were harvested. Cell lysates were subjected to western blotting with the indicated antibodies. The same set of 30 μg of lysates was subjected to western blotting using two membranes; one was probed with anti-HA antibody and then reprobed with anti-α-tubulin antibody; the other was probed with anti-HIV-1 p24 antibody and then reprobed with anti-cyclophilin A antibody. One set of representative data shown in Fig. 1a is shown. (**c**) RT activity in the supernatants used in Fig. 1b was measured and the results are shown as fold-increase compared to the control (0 μg of pHASPSB1). (**d**) Approximately 1.5 × 10^6^ HEK293T cells were co-transfected with 0.4 μg of pHCMV-VSV-G, 0.9 μg of pCMV deltaR8.2 and 1.4 μg of pCSII-CMV-luc-IRES2-Bsd together with either EV or increasing amounts of pHASPSB1. The total amount of the effector plasmids was adjusted to 2.5 μg. Transduction efficiency in MT-4 cells exposed to the culture supernatant was evaluated. The luciferase activity was normalized against the protein concentration and the results are shown as fold-increase compared to the control (0 μg of pHASPSB1). (**e**) Transfected HEK293T cells shown in Fig. 1d were harvested 48 hours post-transfection. Cell lysates were subjected to western blotting as in Fig. 1b. (**f**) The same set of 30 μg of lysates used in (**e**) and virus particles obtained by ultracentrifugation of culture supernatant were subjected to western blotting with the indicated antibodies. (**a**,**d**) Averages and standard errors calculated from three independent experiments are shown. One representative set of western blotting results is shown. Full-length blots are presented in Supplementary Fig. S3. *P < 0.05, ****P < 0.0001.

To determine whether the SPSB proteins have any impact on HIV-1 production, we co-transfected HEK293T cells with the HIV-1 proviral plasmid pNL4-3 and a plasmid expressing one of the HA-tagged SPSB proteins, or with empty vector (EV). We then evaluated HIV-1 production using TZM-bl indicator cells which express firefly luciferase when they are infected with HIV-1. HA-tagged SPSB1, but not SPSB2 or SPSB3, enhanced HIV-1 production (data not shown). HIV-1 production induced by SPSB1 peaked with 2.0 μg of the SPSB1 expression plasmid (pHASPSB1) as revealed by a >4-fold increase over the control reporter gene activity (Fig. 1a). Western blotting studies revealed a plasmid dose-dependent increase in the SPSB1 and Gag (Pr55 and p24) protein levels, ranging from 0.5 μg to 2.0 μg of pHASPSB1 (Fig. 1b), which correlated well with the reporter assay results (Fig. 1a). To assess whether SPSB1 increased virus release from producer cells, we measured the reverse transcriptase (RT) activity in the culture supernatants, which was elevated in the presence of SPSB1 (Fig. 1c). Next, we hypothesized that SPSB1 may enhance the production of lentiviral vector derived from HIV-1. We co-transfected HEK293T cells with a lentiviral transfer vector capable of expressing firefly luciferase (pCSII-CMV-luc-IRES2-Bsd), packaging plasmid (pCMV deltaR8.2) and a plasmid expressing the vesicular stomatitis virus glycoprotein (pHCMV-VSV-G) along with the EV and/or pHASPSB1. MT-4 human T-cells were then exposed to the lentiviral vector-containing culture supernatant of the transfected cells and luciferase activity was measured 24 hours later. As expected from the results of HIV-1 production (Fig. 1a), co-expression of SPSB1 increased the transduction of the lentiviral vector in a dose-dependent manner, ranging from 0.5 μg to 2.0 μg of pHASPSB1 (Fig. 1d). Again, western blotting studies showed an increase in the expression levels of the SPSB1, Gag and VSV-G proteins in producer cells (Fig. 1e,f). Quantification of western blotting signal confirmed concordant increase in Gag and VSV-G protein expression in producer cells as well as elevated incorporation of VSV-G protein into virus particles (Supplementary Fig. S1). These results indicate that ectopic expression of SPSB1 increased the expression of viral structural proteins in producer cells and suggest that this led to enhanced lentivirus production.

### SPSB1 activates the HIV-1 LTR and CMV promoter

**Figure 2:**
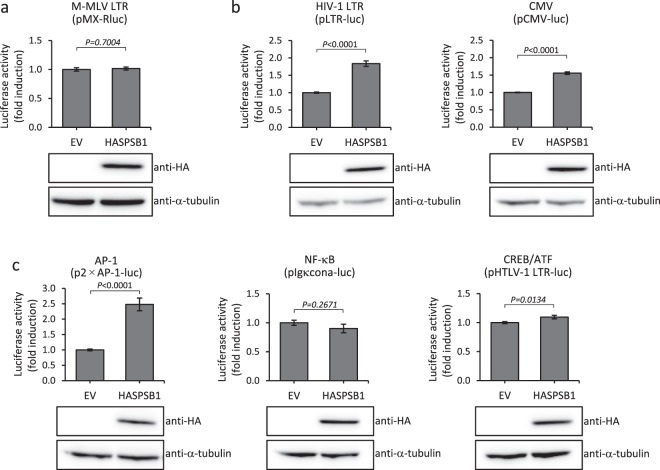
SPSB1 activates the HIV-1 LTR and CMV promoter. (**a**) Approximately 2 × 10^5^ HEK293T cells were co-transfected with 0.1 μg of pMX-Rluc and 0.2 μg of EV or pHASPSB1 (HASPSB1). Transfected cells were harvested 24 hours post-transfection. The luciferase activity was normalized against the protein concentration and the results are shown as fold-increase compared to the EV (upper panel). The bars indicate the standard errors calculated from three independent experiments. Thirty μg of cell lysates were subjected to western blotting with anti-HA or anti-α-tubulin antibodies. (**b**) Approximately 2 × 10^5^ HEK293T cells were co-transfected with 0.1 μg of pLTR-luc (left panel), or pCMV-luc (right panel) together with 0.2 μg of EV or pHASPSB1. pMX-Rluc (0.05 μg) was co-transfected in each sample as an internal control to normalize transfection efficiency. The firefly luciferase activity was normalized to *Renilla* luciferase activity and the results are shown as fold-increase compared to the EV (upper panels). The bars indicate the standard errors calculated from three independent experiments. Thirty μg of cell lysates were subjected to western blotting with anti-HA or anti-α-tubulin antibodies. Full-length blots are presented in Supplementary Fig. S4. (**c**) Approximately 2 × 10^5^ HEK293T cells were co-transfected with 0.1 μg of p2 × AP-1-luc (left panels), pIgκcona-luc (middle panels) or pHTLV-1 LTR-luc (right panels) together with 0.2 μg of EV or pHASPSB1. pMX-Rluc (0.05 μg) was co-transfected in each sample as an internal control to normalize transfection efficiency. The firefly luciferase activity was normalized to *Renilla* luciferase activity and the results are shown as fold-increase compared to the EV (upper panels). The bars indicate the standard errors calculated from three independent experiments. Thirty μg of cell lysates were subjected to western blotting with anti-HA or anti-α-tubulin antibodies. Relative luciferase activities are shown with average and standard error calculated from four independent experiments. One representative set of western blotting results is shown. Full-length blots are presented in Supplementary Fig. S4.

Because the expression of viral structural proteins is controlled by the HIV-1 LTR promoter for HIV-1 production (Fig. 1b) and by the CMV promoter in the packaging plasmid (pCMV deltaR8.2) for lentiviral vector production (Fig. 1d), we assessed whether SPSB1 activates the HIV-1 LTR and CMV promoter. We firstly sought a promoter whose activity is not influenced by SPSB1 expression and so can be used as an internal control in the Dual-Luciferase Reporter Assay. We found that the Moloney murine leukemia virus (M-MLV) LTR promoter is appropriate for this task (Fig. 2a). Reporter gene assays revealed that SPSB1 activated the HIV-1 LTR (Fig. 2b, left panels) and CMV promoter (Fig. 2b, right panels). Transcription factors that contribute to activation of the HIV-1 LTR and CMV but not the M-MLV LTR promoter include AP-1, NF-κB and CREB/ATF^20,21^. We thus hypothesized that SPSB1 enhances transcription via some of these transcription factors. To test this hypothesis, we employed p2 × AP-1-luc^22^, pIgκcona-luc^23^ and pHTLV-1 LTR-luc^23^ reporter plasmids. HTLV-1 LTR is known to be activated by CREB/ATF. SPSB1 activated AP-1-dependent (Fig. 2c, left panels), but not NF-κB-dependent (Fig. 2c, middle panels) or CREB/ATF-dependent luciferase expression (Fig. 2c, right panels) in HEK293T cells. We confirmed the expression of SPSB1 in transfected cells by western blotting (Fig. 2a–c, lower panels). The results suggest that SPSB1 activates the HIV-1 LTR and CMV promoter through AP-1 activation and eventually increases lentivirus production.

### HTLV-1 Tax robustly enhances lentiviral vector production

**Figure 3:**
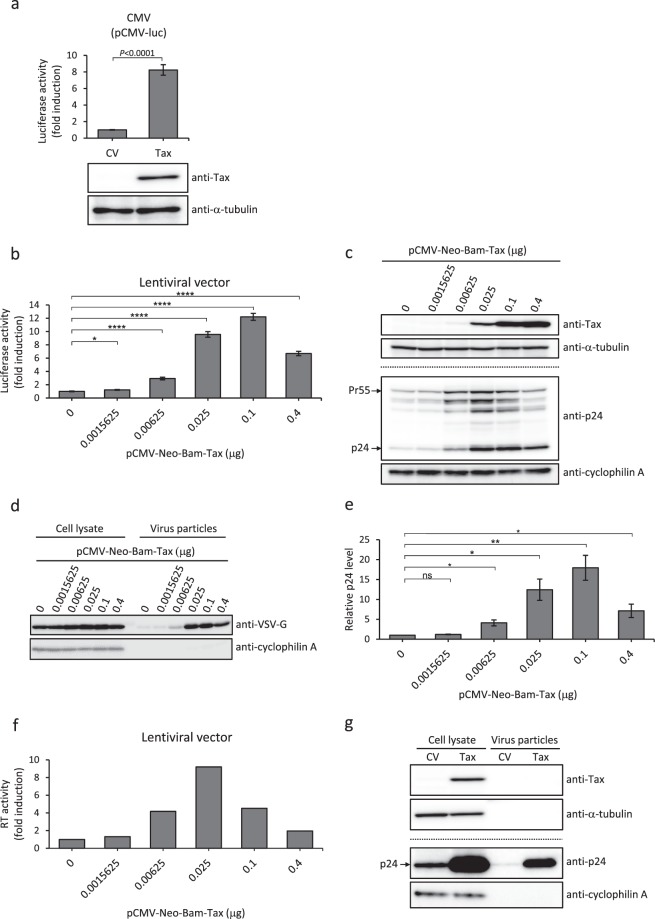
HTLV-1 Tax robustly enhances lentiviral vector production. (**a**) Approximately 2 × 10^5^ HEK293T cells were co-transfected with 0.1 μg of pCMV-luc and 0.2 μg of pCMV-Neo-Bam-Tax (Tax) or pCMV-Neo-Bam as control vector (CV). The luciferase activity was normalized against the protein concentration and the results are shown as fold-increase compared to the CV (upper panel). Cell lysates were subjected to western blotting with the indicated antibodies. (**b**) Approximately 1.5 × 10^6^ HEK293T cells were co-transfected with 0.4 μg of pHCMV-VSV-G, 0.9 μg of pCMV deltaR8.2 and 1.4 μg of pCSII-CMV-luc-IRES2-Bsd together with either CV or increasing amounts of pCMV-Neo-Bam-Tax. The total amount of the effector plasmids was adjusted to 0.4 μg. Lentiviral vector-containing culture supernatants were harvested 48 hours post-transfection. Transduction efficiency in MT-4 cells exposed to the culture supernatant was evaluated. The luciferase activity was normalized against the protein concentration. (**c**) Transfected cells shown in Fig. 3b were harvested. The same set of 30 μg of lysates was subjected to western blotting using two membranes; one was probed with anti-Tax antibody and then reprobed with anti-α-tubulin antibody; the other was probed with anti-HIV-1 p24 antibody and then reprobed with anti-cyclophilin A antibody. One set of representative data shown in Fig. 3b is shown. (**d**) The same set of 30 μg of lysates used in (**c**) and virus particles obtained by ultracentrifugation of each culture supernatant were subjected to western blotting with the indicated antibodies. (**e**) The amounts of p24 protein in the supernatants of transfected cells were quantified with HIV-1 CA (p24) ELISA. (**f**) RT activity in the supernatant used in Fig. 3d was measured and the results are shown as fold-increase compared to the control (0 μg of pCMV-Neo-Bam-Tax). (**g**) Lentiviral particles were collected by ultracentrifugation. Both cell lysate and pelleted virus particles were then subjected to western blotting as in Fig. 3c. (**a**,**b**,**e**) Averages and standard errors calculated from three independent experiments are shown. One representative set of western blotting and RT assay results is shown. Full-length blots are presented in Supplementary Fig. S5. *P < 0.05, **P < 0.01, ****P < 0.0001, ns indicates not significant.

We next hypothesized that proteins that simultaneously activate AP-1, NF-κB and CREB/ATF would augment lentivirus production much more efficiently than SPSB1. One such candidate protein is HTLV-1 Tax, which has been reported to activate a number of cellular transcription factors including AP-1, NF-κB and CREB/ATF^19^. A previous study had reported that Tax activated the CMV promoter in HEK293 cells, but not in HEK293T cells^24^. However, we found that Tax did strongly activate the CMV promoter in HEK293T cells (Fig. 3a). In reporter assays with Tax, we found that the activity of all the tested promoters including the M-MLV LTR promoter was influenced by Tax expression (data not shown), and therefore we normalized the reporter assay results hereafter to protein concentration. Co-expression of Tax in lentiviral vector producer cells achieved an up to 12-fold increase in lentiviral vector production as revealed by luciferase activity in infected cells (Fig. 3b). Western blotting confirmed an increase in Tax, Gag and VSV-G expression in producer cells (Fig. 3c,d) and VSV-G incorporation into lentiviral vector particles (Fig. 3d). Quantification of signal revealed that western blot results correlated well with those of lentiviral vector transduction (Fig. 3b and Supplementary Fig. S2). To assess whether Tax increased virus release from producer cells, we measured the concentration of HIV-1 Gag using a specific ELISA (Fig. 3e) and RT activity (Fig. 3f) in the culture supernatant. The amount of Gag in the supernatant was similarly elevated in the presence of Tax, which correlated well with transduction efficiency and Gag expression levels in producer cells (Fig. 3b,c). RT activity also increased when Tax was co-expressed in producer cells. Finally, from the view point of safety and decontamination, we explored whether Tax is incorporated into lentiviral vector particles. Virus particles in the culture supernatant were collected by ultracentrifugation as pellets through a sucrose cushion. Tax protein was not detectable by western blotting in the pellets obtained by ultracentrifugation (Fig. 3g), suggesting that Tax is unlikely to be incorporated into the lentiviral vector particles.

## Discussion

In this study, we have shown that co-expression of a transcriptional activator such as HTLV-1 Tax greatly improves lentiviral vector production by enhancing the expression of viral proteins in producer cells (Fig. 3). Tax enhanced reporter gene expression driven by the CMV promoter (Fig. 3a), Gag and VSV-G expression in producer cells, p24 amount and RT activity in the culture supernatant, all of which mostly correlated with the results of lentiviral vector transduction (Fig. 3b–f). In our experimental setting, the CMV promoter drives the lentiviral transfer vector (pCSII-CMV-luc-IRES2-Bsd), packaging plasmid (pCMV deltaR8.2) and the plasmid expressing the envelope (pHCMV-VSV-G). Thus, these results strongly suggest that enhanced gene expression from these plasmids resulted in efficient production of the lentiviral vector. However, formally, it may also be possible that Tax activates expression of certain host HIV-1 dependency factors which then contribute to enhanced lentiviral expression. Of note, lentiviral vector production declined from the peak when 2.5 μg of the SPSB1 expression vector (Fig. 1d) or 0.4 μg of the Tax expression vector (Fig. 3b) was co-transfected, cautioning that an excess amount of these plasmids can have adverse effects on lentiviral vector production. Indeed, the expression of Gag and VSV-G in producer cells was reduced under such conditions (Figs 1e,f and 3c,d).

Co-expression of SPSB1 increased AP-1- but not NF-κB- or CREB/ATF-dependent transcription (Fig. 2c), resulting in enhanced protein expression from the CMV and HIV-1 LTR promoters (Fig. 2b). This AP-1 activation by SPSB1 may be explained in part by a recent finding that the SPRY domain of RhTRIM5α is essential for AP-1 but not NF-κB activation^25^. In addition, because ubiquitination of heterogeneous nuclear ribonucleoprotein A1 (HnRNP A1) by SPSB1 induces the expression of Rac1B^26^, which in turn elevates mRNA expression of c-Jun N-terminal kinase 2 and c-JUN^27^, it is plausible that these events cooperate to achieve activation of AP-1-dependent transcription. Because HTLV-1 Tax is a powerful viral *trans*-acting protein, its incorporation into lentiviral vector particles, if sufficient for its *trans*-acting functions, could elicit undesired effects in target cells. Although Tax is unlikely to be present in lentiviral vector particles, at least to the extent that our western blotting assay can show this (Fig. 3g), it may be necessary to further verify this in other ways. We have focused in this paper on the events that facilitate transcription for lentiviral vector production in producer cells, and undoubtedly, this new strategy should further be substantiated in animal studies.

In summary, this study presents an easily available, safe and previously undescribed method for improving lentiviral vector production. This will certainly contribute to down-scaling the costly cell culture and transfection procedures and can be used in a wide variety of *in vitro* and *in vivo* applications by combining it with current concentration and purification techniques.

## Materials and Methods

### Cells

The cell lines HEK293T (Invitrogen Corp., Carlsbad, CA) and TZM-bl [National Institutes of Health (NIH) AIDS Research and Reference Reagent Program] were propagated in Dulbecco’s modified Eagle’s medium (Nacalai Tesque, Inc., Kyoto, Japan) supplemented with 10% fetal bovine serum (FBS), 100 U/ml of penicillin and 100 μg/ml of streptomycin. MT-4 cells were maintained in complete RPMI 1640 medium (Nacalai Tesque) supplemented with 10% FBS, 100 U/ml of penicillin and 100 μg/ml of streptomycin.

### Plasmids

The cDNA encoding SPSB1 was amplified by RT-PCR using HEK293T cell-derived total RNA as template. Primers HA-SPSB1–5 (5′-CCGAATTCCACCATGTACCCATACGACGTCCCAGACTACGCTGGTCAGAAGGTCACTGGAGGGATCA-3′) and HA-SPSB1–3 (5′-GCCTCGAGTCACTGGTAGAGGAGGTAGGC-3′) were used for SPSB1 cDNA amplification. The amplified cDNA was inserted into the *Eco*RI and *Xho*I sites of pIRES-PURO (Clontech Laboratories, Mountain View, CA), and sequenced. The resultant plasmid is referred to as pHASPSB1. pNL4-3^28^ was obtained from NIH AIDS Research and Reference Reagent Program. To generate pCMV-luc, the coding sequence of firefly *luciferase* was amplified by PCR using pGL3-Basic (Promega Corp., Madison, WI) as the template. Primers firefly luciferase-5 (5′-GCCTCGAGCCACCATGGAAGACGCCAAAAAC-3′) and firefly luciferase-3 (5′-CCGGATCCTTACACGGCGATCTTTCCGCCC-3′) were used for this PCR. The amplified DNA fragment was inserted into the *Xho* I and *Bam*H I sites of pcDNA3.1(−) (Invitrogen Corp). pCSII-CMV-MCS-IRES2-Bsd was provided by the RIKEN BRC through the National Bio-Resource Project of the MEXT, Japan. To generate pCSII-CMV-luc-IRES2-Bsd, pCSII-CMV-MCS-IRES2-Bsd and pCMV-luc were digested with *Xho* I and *Not* I. The *Xho* I-*Not* I fragment carrying the firefly *luciferase* gene from pCMV-luc was then inserted into the *Xho* I-*Not* I site of pCSII-CMV-MCS-IRES2-Bsd. The resulting plasmid was designated pCSII-CMV-luc-IRES2-Bsd. pHCMV-VSV-G was a kind gift of Dr. I.S.Y. Chen. To construct pMX-Rluc, the coding sequence of *Renilla luciferase* was amplified by PCR using pGL4.84[hRlucCP/Puro] (Promega Corp.) as a template. Primers Renilla luciferase-5 (5′-CCGGATCCGCCACCATGGCTTCCAAG-3′) and Renilla luciferase-3 (5′-AACTCGAGTTAGACGTTGATCCTGGCG-3′) were used for the PCR. The amplified DNA fragment was inserted into the *Bam* HI and *Xho* I sites of pMX^29^. pCMV deltaR8.2, pCMV-Neo-Bam-Tax, pCMV-Neo-Bam, p2 × AP-1-luc, pIgκcona-luc, pHTLV-1 LTR-luc and pLTR-luc were described previously^22,23,30–33^.

### Western blotting

Cell lysates were prepared using lysis buffer (25 mM Tris pH 7.8, 8 mM MgCl_2_, 1 mM DTT, 1% Triton-X 100, 15% glycerol). Protein concentration was determined by the Bradford assay. Proteins were separated by SDS-PAGE, transferred to polyvinylidene difluoride (PVDF) membranes and reacted with a rat monoclonal antibody to the influenza virus haemagglutinin (HA) (3F10, Roche Applied Science, Mannheim, Germany), mouse monoclonal antibody to Tax (MI73)^34^, mouse monoclonal antibody to HIV-1 p24 (39/5.4 A, Abcam, Inc., Cambridge, MA), mouse monoclonal antibody to VSV-G (P5D4)^35^, rabbit polyclonal antibody to cyclophilin A (BML-SA296, Enzo Life Sciences, Inc., Farmingdale, NY) or mouse monoclonal antibody to α-tubulin (DM1A, Sigma-Aldrich Co., St. Louis, MO). Membranes were then incubated with horseradish peroxidase-conjugated rabbit anti-mouse IgG (A206PS, American Qualex International Inc, San Clemente, CA) or goat anti-rat IgG (sc-2006, Santa Cruz Biotechnology, Dallas, TX) and proteins were visualized by western Lightning Plus-ECL (PerkinElmer, Waltham, MA).

### Preparation of virus stocks

Approximately 1.5 × 10^6^ of HEK293T cells cultured in a 6-well plate were co-transfected with 1 µg of pNL4-3 and pHASPSB1 ranging from 0 to 2.5 µg using polyethylenimine (PEI). VSV-G envelope-pseudotyped lentiviral vector carrying the firefly *luciferase* reporter gene was prepared by transfection of HEK293T cells using PEI with 1.4 µg of the lentiviral vector plasmid (pCSII-CMV-luc-IRES2-Bsd), 0.9 µg of a packaging plasmid (pCMV deltaR8.2), 0.4 µg of a VSV-G protein-expression plasmid (pHCMV-VSV-G) and pHASPSB1 ranging from 0 to 2.5 µg. For production of the lentiviral vector in the presence of Tax, HEK293T cells were co-transfected, using PEI, with 1.4 µg of pCSII-CMV-luc-IRES2-Bsd, 0.9 µg of pCMV deltaR8.2 and 0.4 µg of pHCMV-VSV-G together with pCMV-Neo-Bam-Tax and/or pCMV-Neo-Bam. Viruses in the supernatant were harvested 48 hours post-transfection. The amount of HIV-1 capsid (CA) in the supernatant was quantified by HIV-1 CA (p24) enzyme-linked immunosorbent assay (ELISA) (ZeptMetrix Corporation, Buffalo, NY). The method for measurement of RT activity was described previously^36^.

### HIV-1 titration

Approximately 2 × 10^4^ TZM-bl cells in a 48-well plate were exposed to an equal volume of each supernatant containing virus. Cells were harvested and lysed 72 hours post-infection with lysis buffer (25 mM Tris pH 7.8, 8 mM MgCl_2_, 1 mM DTT, 1% Triton-X 100, 15% glycerol). The luciferase activity was measured using the GloMax-Multi Detection system (Promega Corp.).

### Luciferase reporter assay

Approximately 2 × 10^5^ HEK293T cells in a 24-well plate were co-transfected with pLTR-luc, pCMV-luc, pHTLV-1 LTR-luc, pIgκcona-luc or p2 × AP-1-luc together with the effector plasmids using FuGENE6 (Promega Corp.). pMX-Rluc was co-transfected in each sample as an internal control to normalize transfection efficiency for experiments shown in Fig. 2b,c. Transfected cells were harvested and lysed 24 hours post-transfection with lysis buffer (25 mM Tris pH 7.8, 8 mM MgCl_2_, 1 mM DTT, 1% Triton-X 100, 15% glycerol). The firefly and *Renilla* luciferase activities were measured using Dual-Luciferase Reporter Assay System (Promega Corp.) according to the manufacturer’s protocol and the GloMax-Multi Detection system (Promega Corp.). In Figs 1a,d, 2a and 3a,b, the activity of luciferase was normalized against protein concentration determined by the Bradford assay.

### Purification of lentiviral vectors

Two ml of 20% sucrose solution was placed at the bottom of model SW55 ultracentrifuge tubes and overlaid with 2 ml of lentiviral vector-containing culture supernatant. Samples were then centrifuged for 60 minutes at 35,000 rpm at 4 °C. The pelletable fraction was resuspended in PBS (-) and subjected to western blotting.

### Statistical analysis

All statistical analyses were performed using GraphPad Prism 6 software (GraphPad Software, inc, La Jolla, CA). One-way analysis of variance (ANOVA) with Dunnett’s multiple comparison test was used for the data shown in Fig. 1a,d to test whether the means of multiple groups were significantly different from a single group. Significant difference between the two groups was determined using unpaired two-tailed Student’s *t* test (Figs 2a–c and 3a,b,e). *P* values < 0.05 were considered statistically significant.

## Electronic supplementary material

Figure S1, S2, S3, S4 and S5
