# Supplementary material for: Robust Enhancement of Lentivirus Production by Promoter Activation
Source: Sci Rep. 2018 Oct 11;8:15036. doi: 10.1038/s41598-018-33042-5 (PMC6181906; doi:10.1038/s41598-018-33042-5)
Supplement: Supplementary file 1 — Figure S1, S2, S3, S4 and S5 [file 41598_2018_33042_MOESM1_ESM.pdf]

Supplementary information

Robust Enhancement of Lentivirus Production by Promoter Activation

Naoto Suzuki[1], Takeshi Yoshida[1*], Hiroaki Takeuchi[1], Ryuta Sakuma[1,2], Sayaka Sukegawa[1,3], Shoji Yamaoka[1*]

1. Department of Molecular Virology, Tokyo Medical and Dental University (TMDU), Tokyo, Japan
2. Present address: Medical Affairs Unit, ViiV Healthcare K.K. 1-8-1 Akasaka, Minato-ku, Tokyo 107-0052, Japan
3. Present address: Department of Infectious Diseases and Immunology, Clinical Research Center, National Hospital Organization Nagoya Medical Center, Nagoya, Japan.

*correspondence: Department of Molecular Virology, Tokyo Medical and Dental University (TMDU), 1-5-45 Yushima, Bunkyo-ku, Tokyo 113-8519 Japan,
E-mail address: takeshi-yoshida@umin.ac.jp (T.Y.), shojmmb@tmd.ac.jp (S.Y.)

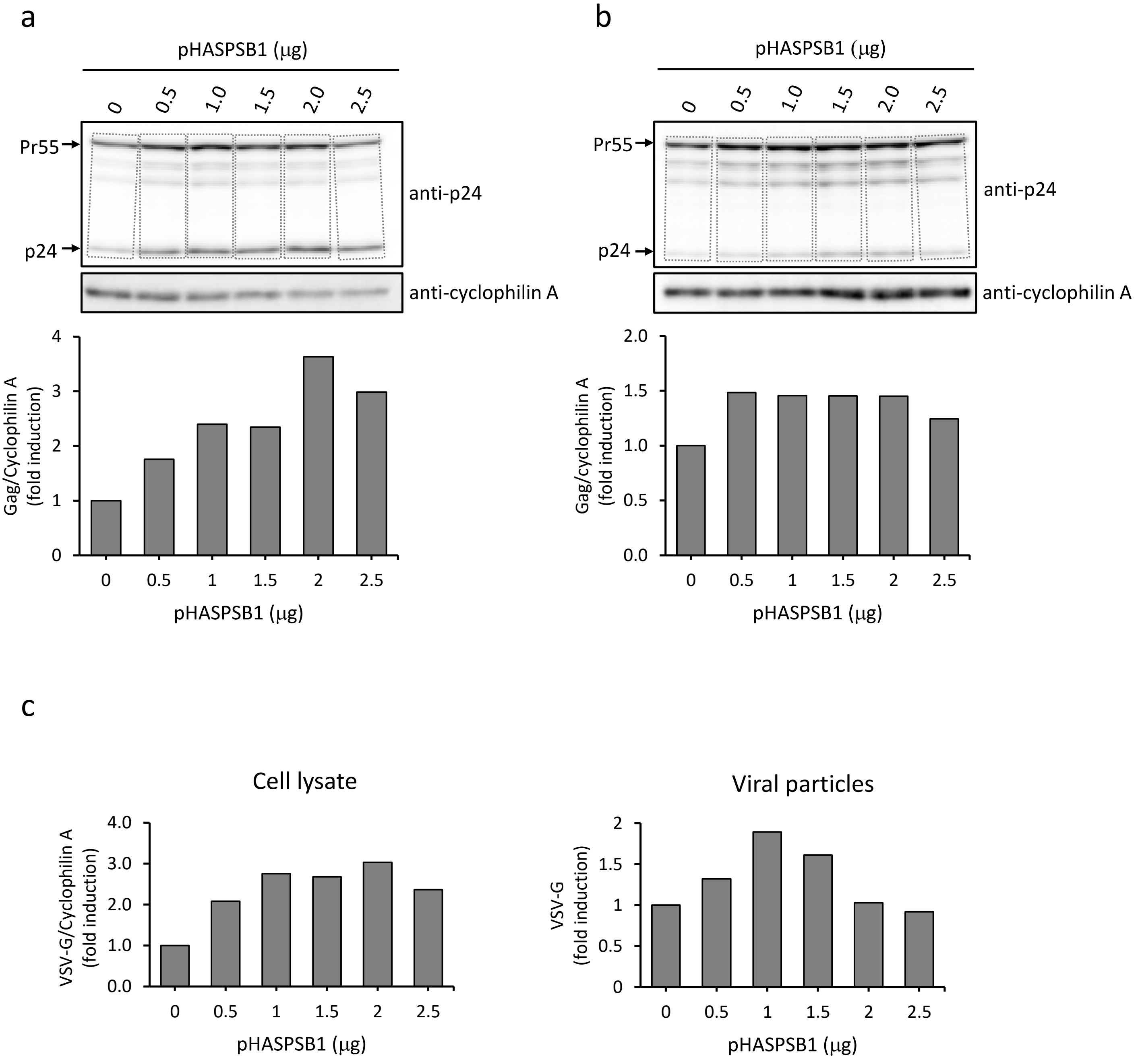

Supplementary Figure S1. Quantification of signal shown in Figure 1b (**a**), 1e (**b**) and 1f (**c**). (**a**, **b**) The expression levels of the Gag protein were quantified and normalized against that of cyclophilin A. The results are shown as fold-increase compared to the control (0 μg of pHASPSB1). The signal in the cropped parts indicate by the gray dashed lines was quantified. (**c**) The expression levels of the VSV-G protein in producer cells were quantified and normalized against that of cyclophilin A. The expression levels of the VSV-G protein in viral particles were quantified. The results are shown as fold-increase compared to the control (0 μg of pHASPSB1).

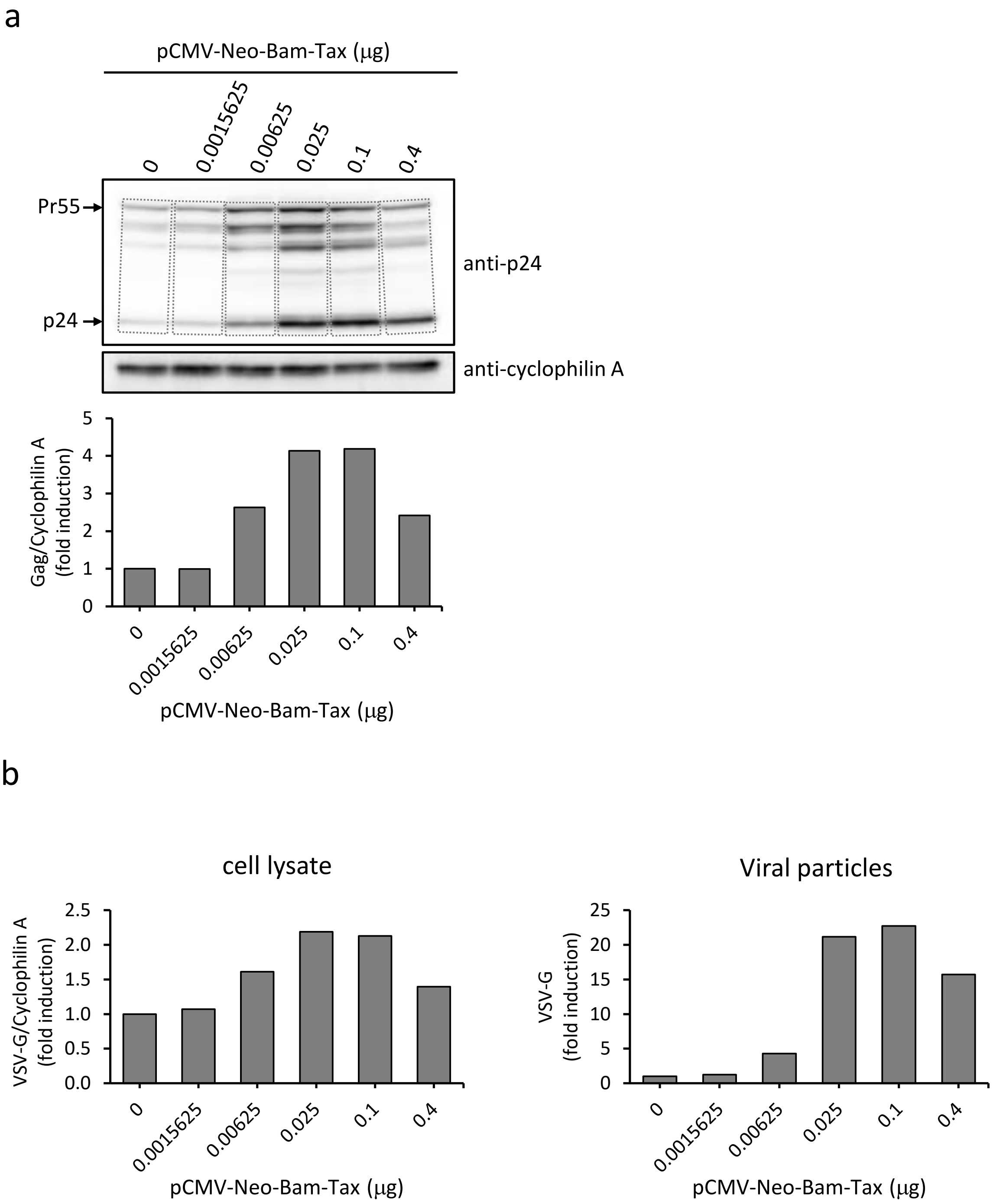

Supplementary Figure S2. Quantification of signal shown in Figure 3c (**a**) and 3d (**b**). (**a**) The expression levels of the Gag protein were quantified and normalized against that of cyclophilin A. The results are shown as fold-increase compared to the control (0 μg of pCMV-Neo-Bam-Tax). The signal in the cropped parts indicate by the gray dashed lines was quantified. (**b**) The expression levels of the VSV-G protein in producer cells were quantified and normalized against that of cyclophilin A. The expression levels of the VSV-G protein in viral particles were quantified. The results are shown as fold-increase compared to the control (0 μg of pCMV-Neo-Bam-Tax).

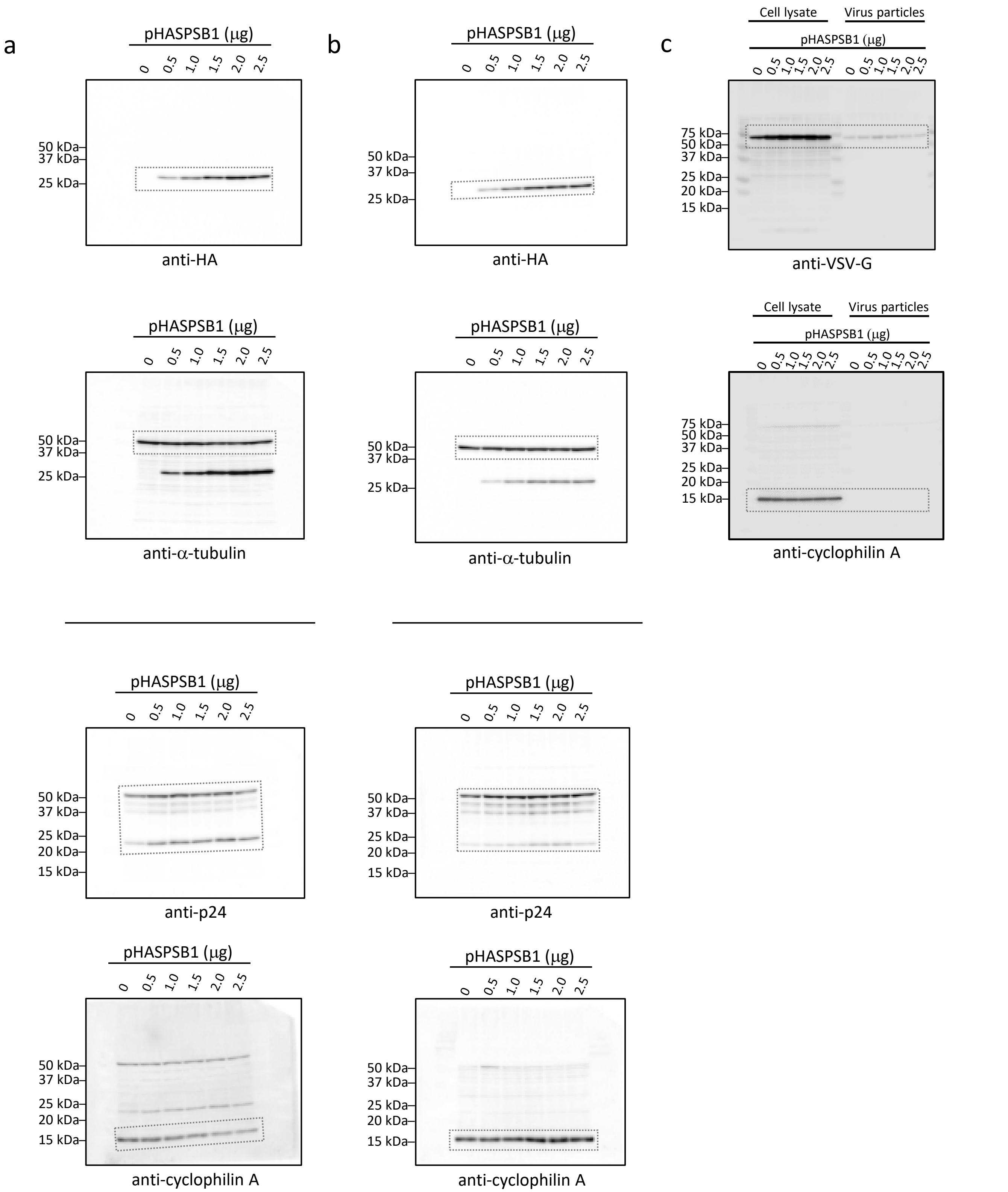

Supplementary Figure S3. Full-length blots of western blotting shown in Figure 1b (**a**), 1e (**b**) and 1f (**c**). (**a, b**) The same set of 30 μg of lysates was subjected to western blotting using two membranes; one was probed with anti-HA antibody and then reprobed with anti-α-tubulin antibody; the other was probed with anti-HIV-1 p24 antibody and then reprobed with anti-cyclophilin A antibody. (**c**) Lysates were subjected to western blotting with anti-VSV-G antibody. Anti-cyclophilin A antibody was used as a loading control. The cropped parts indicate by the gray dashed lines are shown in Figure 1b (**a**), 1e (**b**) and 1f (**c**).

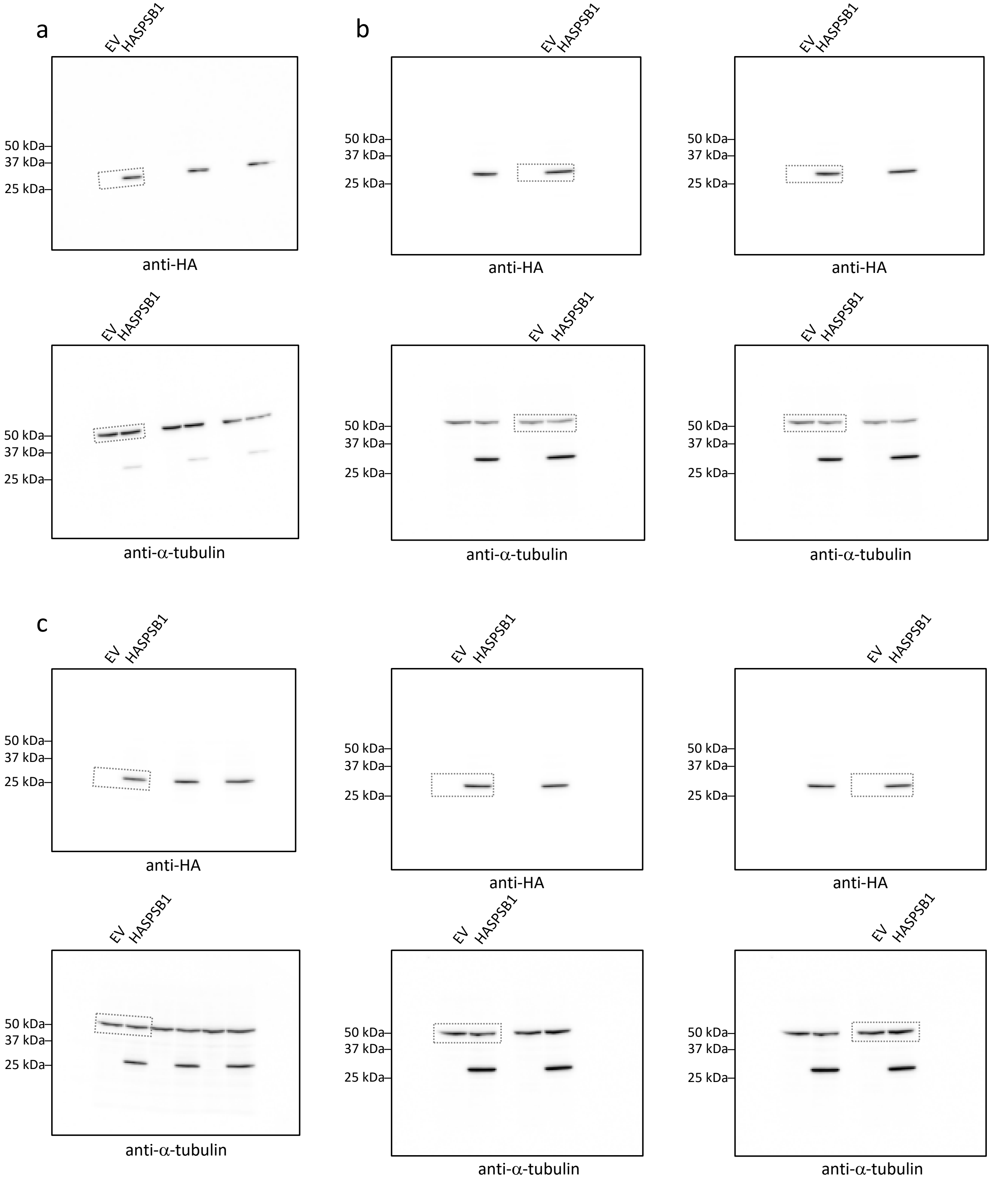

Supplementary Figure S4. Full-length blots of western blotting shown in Figure 2a (**a**), 2b (**b**) and 2c (**c**). (**a**, **b, c**) Thirty μg of lysates were subjected to western blotting with anti-HA antibody. Anti-α-tubulin antibody was used as a loading control. The cropped parts indicate by the gray dashed lines are shown in Figure 2a (**a**), 2b (**b**) and 2c (**c**).

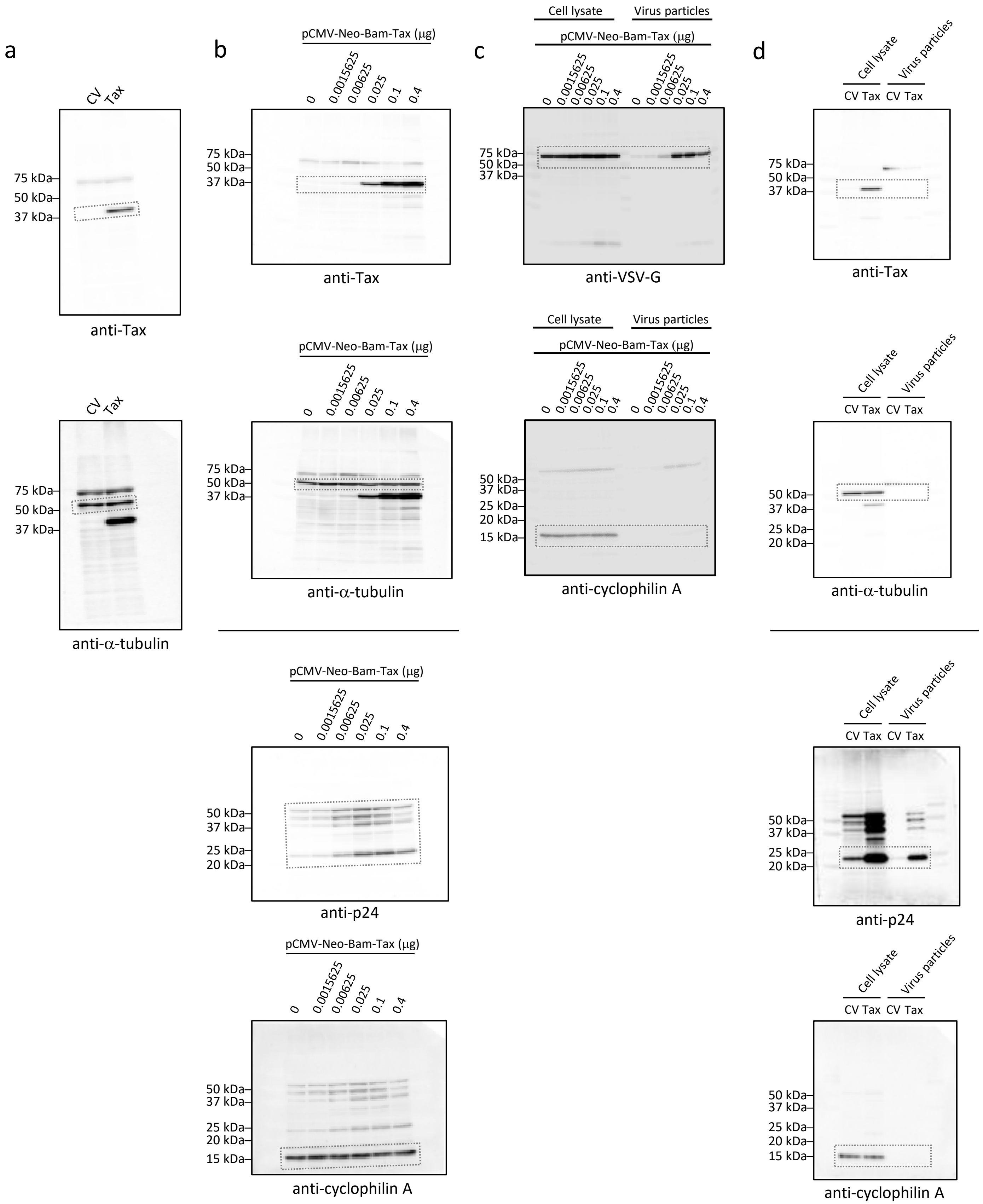

Supplementary Figure S5. Full-length blots of western blotting shown in Figure 3a (**a**), 3c (**b**), 3d (**c**) and 3g (**d**). (**a**) Thirty μg of lysates were subjected to western blotting with anti-HTLV-1 Tax antibody. Anti-α-tubulin antibody was used as a loading control. The cropped parts indicated by the gray dashed lines are shown in Figure 3a. (**b, d**) The same set of 30 μg of lysates was subjected to western blotting using two membranes; one was probed with anti-HTLV-1 Tax antibody and then reprobed with anti-α-tubulin antibody; the other was probed with anti-HIV-1 p24 antibody and then reprobed with anti-cyclophilin A antibody. (**c**) Lysates were subjected to western blotting with anti-VSV-G antibody. Anti-cyclophilin A antibody was used as a loading control. The cropped parts indicate by the gray dashed lines are shown in Figure 3a (**a**), 3c (**b**), 3d (**c**) and 3g (**d**).
